# A Novel Transport Mechanism for MOMP in *Chlamydophila pneumoniae* and Its Putative Role in Immune-Therapy

**DOI:** 10.1371/journal.pone.0061139

**Published:** 2013-04-24

**Authors:** Francis O. Atanu, Ernesto Oviedo-Orta, Kimberly A. Watson

**Affiliations:** 1 School of Biological Sciences, Whiteknights Campus, University of Reading, Reading, Berkshire, United Kingdom; 2 University of Surrey, Faculty of Health and Medical Sciences, Guildford, United Kingdom; Russian Academy of Sciences, Institute for Biological Instrumentation, Russian Federation

## Abstract

Major outer membrane proteins (MOMPs) of Gram negative bacteria are one of the most intensively studied membrane proteins. MOMPs are essential for maintaining the structural integrity of bacterial outer membranes and in adaptation of parasites to their hosts. There is evidence to suggest a role for purified MOMP from *Chlamydophila pneumoniae* and corresponding MOMP-derived peptides in immune-modulation, leading to a reduced atherosclerotic phenotype in apoE^−/−^ mice via a characteristic dampening of MHC class II activity. The work reported herein tests this hypothesis by employing a combination of homology modelling and docking to examine the detailed molecular interactions that may be responsible. A three-dimensional homology model of the *C. pneumoniae* MOMP was constructed based on the 14 transmembrane β-barrel crystal structure of the fatty acid transporter from *Escherichia coli*, which provides a plausible transport mechanism for MOMP. Ligand docking experiments were used to provide details of the possible molecular interactions driving the binding of MOMP-derived peptides to MHC class II alleles known to be strongly associated with inflammation. The docking experiments were corroborated by predictions from conventional immuno-informatic algorithms. This work supports further the use of MOMP in *C. pneumoniae* as a possible vaccine target and the role of MOMP-derived peptides as vaccine candidates for immune-therapy in chronic inflammation that can result in cardiovascular events.

## Introduction

*Chlamydophila pneumoniae* is an obligate Gram negative bacterium responsible for approximately 20% of community acquired lower respiratory disease [1] and for at least 10% of community acquired pneumonia [2], [3]. The Gram negative bacterium, belonging to the genus *Chlamydophila*, includes other members of the genus such as *C. psittaci* and *C. trachomatis,* all of which are responsible for human infections. *C. pneumoniae* infections are also associated with bronchitis, sinusitis and other acute and chronic inflammatory diseases such as atherosclerosis [4]. Atherosclerosis is a chronic multifactorial inflammatory disease including genetic and environmental factors and long-term bystander inflammation by pathogenic microorganisms such *Cytomegalovirus* and *Herpes virus* [5]. This association has been confirmed by studies on the immune-histochemical properties and DNA fingerprinting studies on atherosclerotic plaques both in human and animal models of the disease [6], [7]. Some results have shown the repeated presence of *Chlamydophila pneumoniae* footprints in atherosclerotic tissues and a near to absence in adjoining normal tissues. It has been suggested that *Chlamydophila pneumoniae* potentiates inflammation/development of atherosclerosis by at least one of the following mechanisms; induction of matrix metalloproteinases [8], induction of macrophages to secrete adhesion molecules, cytokines [9], [10], enhancing oxidative remodelling of low density lipoprotein [9] and calcification of atherosclerotic plaques [11]. We have recently suggested another mechanism which involves the dampening of anti-Chlamydophila antibody and T cell responses [12].

Gram negative bacteria are characterised by their possession of two lipid bilayer membranes separated by a periplasmic layer. The outer membrane of Gram negative bacteria has an additional layer of lipopolysaccharide. This membrane system is a defensive barrier against the diffusion of lipophilic or otherwise hydrophilic xenobiotics, which may pose danger to the survival of the bacteria [13]. To enhance the uptake of solutes from the extracellular environment, Gram negative bacteria have evolved outer membrane proteins (OMP) for this purpose. In *Chlamydophila pneumoniae*, the majority of outer membrane protein present is a 40 kDa protein constituting about 60% of the total mass of its outer membrane and hence it is referred to as the major outer membrane protein (MOMP) [14], [15]. MOMP, in the genus *Chlamydophila*, is relatively conserved and typified by several cysteine residues. These cysteine residues contribute to the formation of disulphide bridges which act to stabilise the membrane integrity mainly in elementary bodies. Typical of most outer membrane proteins of Gram negative bacteria, these form β-barrel structures in the outer membrane in a fashion identical to their orthologs in the mitochondria and choloroplasts [16], [17]. The importance of the transmembrane β-barrel is exemplified by the fact that approximately 2–3% of the genome of Gram negative bacteria encode for this class of protein [18]. However, at the time of writing this paper, of the 80, 550 non-redundant protein structures in the Protein Data Bank (www.pdb.org) only 308 (http://blanco.biomol.uci.edu/mpstruc/listAll/list) represent membrane protein structures and of these only 125 are represented by β-barrel folds. This gap is a result of the tremendous challenges that continue to accompany structure determination of membrane protein structures. Therefore, *in silico* prediction of three-dimensional structure remains a valuable tool for understanding the role of these proteins in biology and medicine.

Preliminary evidence suggests that the purified MOMP of *Chlamydophila pneumoniae* has anti-atherosclerotic and anti-inflammatory effects [12], [19]–[21]. This mechanism could potentially favour Chlamydophila’s survival within macrophages while keeping a slow chronic induction of inflammation within atherosclerotic plaques contributing to their development over many years. These results also point to the involvement of MHC class II peptide regulation of inflammation-linked to atherosclerosis development in an apoE^−/−^ mice model. As such, MHC class II peptides represent potential vaccine candidates for the design of an anti-atherosclerosis vaccine.

Molecular modelling of peptide-MHC complexes provides an insight into the detailed interactions stabilising these complexes. Such methods rely on empirical algorithms for the prediction of the orientation of the peptides and subsequent binding affinity for specificity pockets within the binding site [22], [23]. The complexity of molecular docking approaches range from rigid body docking to flexible docking of ligands to their receptors. The computational results provided by these techniques can provide useful information regarding the most promising candidates for further investigation and can be an important factor in early elimination of poor lead candidates.

Herein we show, *in silico* algorithms used to construct the first three dimensional homology model of MOMP from *Chlamydophila pneumoniae* that may support previously formulated hypothesis on its mechanism of action and the potential molecular interactions of MOMP-derived peptides bound to MHC class II receptors. The data presented in this work provides new insights into further understanding of the role of MOMP and MOMP-derived peptides in the pathogenesis of inflammatory diseases.

## Materials and Methods

All calculations and analyses were performed using a Windows 7 Enterprise operating system; AMD Phenom™II X6 1045T Processor 2.70 GHz, and 4.00 GB RAM memory. Unless otherwise stated, all other figures were prepared using PyMOL [24].

### Sequence Analysis

Blastp was used to find the homologs of MOMP and multiple sequence alignments were performed using ClustalW [25], using default settings and resulting sequence identities (%) and similarities (%) were calculated manually. Phylogenetic plots were rendered using TreeView [26].

### Homology Modelling

Two template-based structure prediction methods were employed, Phyre [27] and IntFold [28], in the search for a suitable homology model template for MOMP. Amino acid sequences for the chosen template (P10384 and P77697 for the fatty acid transporter protein) and the target (P27455, Q6LDD2 and Q9JQF6 for MOMP) were obtained from UniProt [29]. MODELLER [30] was used to build a homology model of MOMP, based on the 2.60 Å resolution fatty acid transporter crystal structure, as suggested by IntFold. Any bound ligands in the crystal structure of the template, such as lauryl dimethylamine-n-oxide, (hydroxyethyloxy)tri(ethyloxy)octane and copper ions, were removed during modelling.

### Statistical Analysis and Validation of MOMP Homology Models

All homology models were analysed for optimal geometric characteristics using Procheck [31], What_Check [32], Verify 3D [33], [34], Errat [35] and Prove [36] found at the SAVeS server (http://nihserver.mbi.ucla.edu/SAVES/). RMSDs between the model and the template were calculated by TM-align [37] and selection of the final model was made based on comparison of optimal geometric characteristics and agreement with secondary structure predictions, as rendered by TMBB-DB [38].

### Flexible Molecular Docking

Docking experiments were conducted to probe the interactions between four MOMP-derived 15-mer novel peptides and the MHC class II family of antibodies, specifically I-Ab and HLA DRB1*0401, two alleles strongly linked to inflammatory diseases. The four MOMP-derived peptides (Table 1) were derived using epitope prediction algorithms[39]–[43] capable of predicting MHC class II restricted peptides, based on the MOMP sequence, and assessed for their ability to be recognised by murine MHC class II molecules. Each of the four peptides were modelled using the Biopolymer application as provided by the software package Sybyl-X 1.3 as β-strands, hydrogens were added in idealised geometry and subsequent minimisation of bond torsion as well as whole molecule was performed using the conjugate gradient algorithm [44]. Two MHC protein structures were retrieved from the Protein Data Bank (PBD codes 1MUJ and 2SEB). IMUJ (I-Ab) is the mouse MHC protein complexed with a Class II-associated invariant chain peptide (CLIP), while 2SEB (HLA DRB1*0401) represents the human allele protein bound to a peptide fragment of human collagen II. Non-protein moieties, such as water and N-acetyl glucosamine, were removed prior to docking and hydrogens were added in idealised geometries. Protomol generation and definition of the ligand binding groove was performed using a ligand directed method, which allows the docking of ligands into predefined sites as defined by occupancy of co-crystallised ligands. An overall energy minimisation of each MHC was performed using the Tripos force field [45] and employing a conjugate gradient algorithm [44] with a convergence criterion of 0.05 kcal/mol A^−1^.

**Table 1:** Analysis of the physico-chemical properties of four novel MOMP-derived peptides.

Peptide	Sequence	Grand averagehydropathy (GRAVY)	Aliphatic index	Averagehydro-philicity	% hydrophilic residue composition
**MdP1**	DPSLLIDGTIWEGAA	0.327	117.330	−0.2	27
**MdP2**	KLLKSALLSAAFAGS	0.973	130.670	−0.3	33
**MdP3**	SLSYRLNSLVPYIGV	0.620	142.670	−0.7	33
**MdP4**	DNIRIAQPKLPTAVL	0.107	136.670	0.0	33

Docking studies were performed using the program Surflex-Dock (SFXC, [46]) as provided by Sybyl-X 1.3. Independent docking runs were performed for each of the four peptides against each of the two MHC II protein targets. The Surflex-X docking algorithm docks a given ligand to a receptor using a flexible ligand and a semi-flexible receptor; in this case the peptides were allowed to be fully flexible while the receptor was semi-flexible. This allows for optimisation of potentially favourable molecular interactions, such as those defined by hydrogen bond and van der Waal forces.

The docking results yield a docking score, which takes into consideration entropic, polar, hydrophobic, repulsive and desolvation factors. The score is expressed as total docking score defined by –log_10_Kd, where Kd is the dissociation constant. The free energy of binding of the ligand to the protein was extrapolated from equation (1).
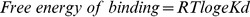
(1)


The molecular interactions of the docked peptides were analysed by CONTACTS, as provided in the CCP4 suite of programs [47]. The MOLCAD module, as provided within Sybyl-X 1.3 program, was used for identification of the surface characteristics (electrostatic, lipophilic) in the binding groove of the target protein.

### Validation of the Docking Procedure

To validate the accuracy of the docking procedure, the original peptide ligands (and all ligands) were removed from the structure file and subsequently docked into the corresponding crystal structure of the MHC proteins, but not using a ligand-directed approach. The resulting “freely docked” (i.e. assuming no prior orientation in the receptor MHC) peptide-MHC complexes were compared to the original peptide-MHC complex, as seen in the original crystal structure of the complex. In this way, the method was validated for its reliability to reproduce essential molecular interactions. Comparative structural orientation of the peptide was calculated as an RSMD between the docked peptide and the original peptide as found in the crystal structure, using TM-align [37].

### Prediction of Peptide-MHC Class II Binding

The binding mode of each peptide was predicted using immuno-informatics prediction servers. Each 15-mer peptide was submitted to the following servers, using default parameters: NetMHC IIpan [48], SMM align [49], NN align [40], Sturnolio [50], RANKPEP [51], MHC2PRED (http://www.imtech.res.in/raghava/mhc2pred/), PROPRED [42], and SVMHC [52] and analysed to derive a consensus nonamer for the binding core.

## Results

### Sequence Analysis

**Figure 1:**
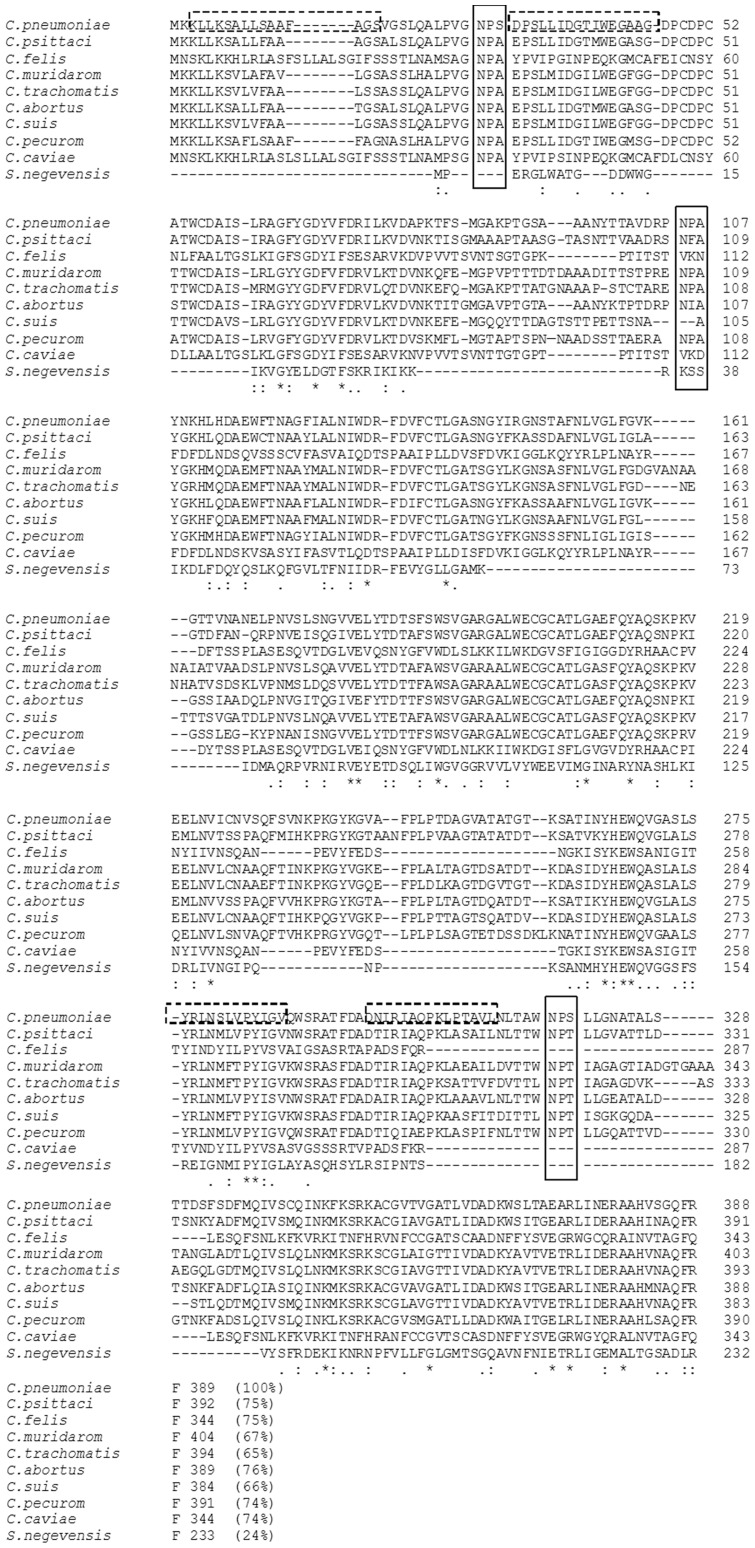
Multiple sequence alignment. ClustalW alignment of the protein sequences of the major outer membrane protein (MOMP) from *C. pneumoniae* and members of the genus *Chlamydophila.* The solid boxes show the motifs putatively involved in substrate transport and selection. The pairwise percentage identity, to MOMP from *C. pneumoniae,* is indicated in parenthesis for each sequence. The dashed boxes show the four MOMP-derived peptides, as predicted using on-line epitope mapping prediction servers.

In an effort to identify suitable homologs of MOMP for homology modelling, a BlastP search was performed using the NCBI databases (http://www.ncbi.nlm.nih.gov/blast/). The search revealed that MOMP had very high sequence identity to other *Chlamydophila* MOMP sequences whose three dimensional structure have yet to be resolved (Figure 1). The NPS motif, located five amino acids from the N-terminus of the mature MOMP protein from *C. pneumonia,* is substituted by NPA in every other *Chlamydophila* species. A second NPS motif, 294 amino acid positions from the N-terminus, is replaced by an NPT motif in all *Chlamydophila* species except *C.caviae* and *C.felis,* which show deletions in the corresponding regions. However, the NPA at position 82 is conserved in *C.muridarom*, *C.trachomatis* and *C.pecurom*. Additionally, the proline residue, in the second position of the NPA motif of MOMP, is substituted by phenylalanine and isoleucine in *C. psittaci* and *C.abortus,* respectively. Phylogenetic analysis reveals that among the genus *Chlamydophila*, MOMP from *C. pneumoniae* is more closely related to those of *C. psittaci* and *C. abortus* (Figure S1). This is reflected also in the higher sequence identity of 75% and 76%, respectively, as compared to MOMPs from the other species (Figure 1).

Interestingly, a standard Blastp search did not identify non-*Chlamydophila* homologs of known structure exhibiting a β-barrel fold expected for MOMP [53]. The top ranking non-*Chlamydophila* homolog identified by the search was the resuscitation promoting factor RpfB (PDB codes 3EO5, 1XSF) from *Mycobacterium tuberculosis* sharing 41% identity with MOMP, which shows a multi-domain structure more characteristic of cell adhesion proteins. This result did not suggest consistency with the known physico-chemical properties of MOMP and indeed other outer membrane proteins from *Chlamydophila*.

As an alternative approach, structure based template prediction methods were utilised (IntFold and Phyre) to identify suitable structural homology templates. Sequences of predicted templates were screened by comparative analysis of MOMP, using ClustalW. Guided by the two NPS motifs and one NPA motif in MOMP, and the overall sequence identity to the predicted templates, selection of a final structural template for modelling was made. The NPA and NPS motifs are common to aquaporins and bacterial fatty acid transporters. The phylogenetic analysis of *Chlamydophila* MOMP with bacterial fatty acid transporters, aquaporins and aquaglyceroporins, as performed herein, shows the uniqueness of MOMP and its unanticipated relatedness to non-prokaryotic NPA motif bearing proteins (Figure S1).

### Homology Modelling

The structure based template method Phyre produced two possible template structures, PDB codes 2X27 and 2F1T, corresponding to OM Oprg from *Pseudomonas aeruginosa* and OmpW from *Escherichia coli*, respectively. Both 2X27 and 2F1T exhibit an 8-stranded β-barrel structure but share less than 15% identity and, only 34.3% and 26.4% sequence similarity, respectively, compared with the MOMP sequence. A structural template for MOMP was found by IntFold, based on the crystal structure of the fatty acid transporter FadL from *Escherichia coli* (PBD code 1T16). In this case, the sequence identity was 16.9% and the sequence similarity was 39.6%. This fatty acid transporter has an NPA motif within the first 30 residues of its primary structure, analogous to MOMP.

**Figure 2:**
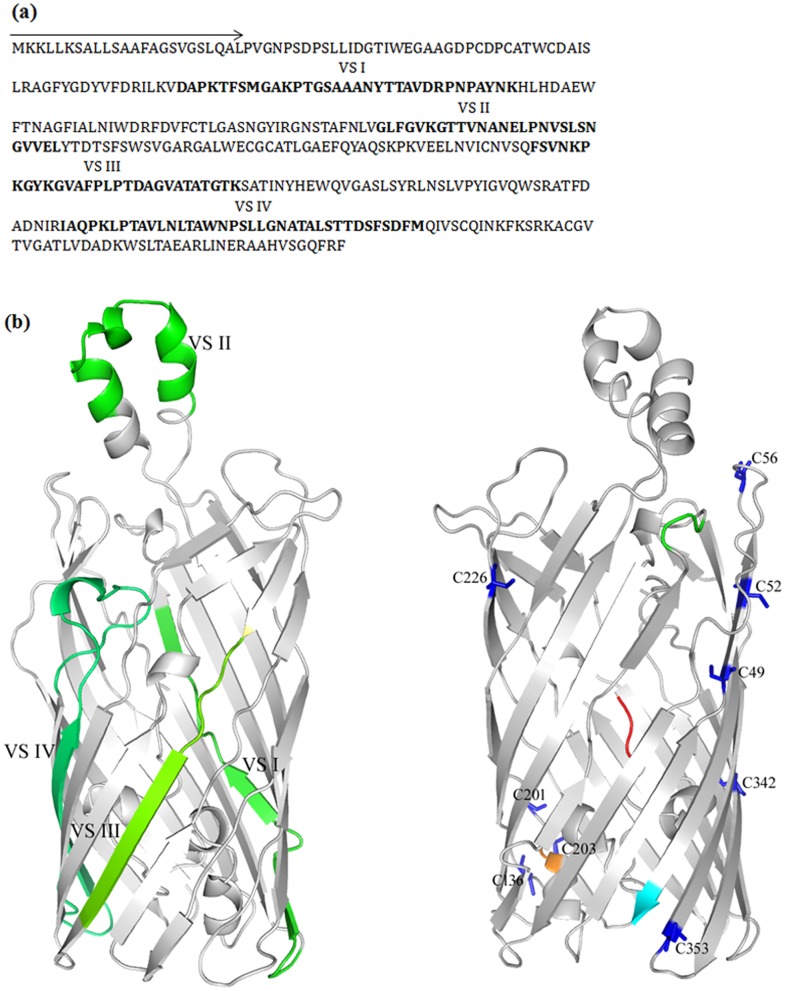
MOMP sequence analysis and homology model. (a) Primary sequence of MOMP from *C. pneumoniae*. The signal sequence is indicated by an arrow while the four variable segments (VS), interspersed between the five constant domains, are highlighted in bold. (b) *Left* - Cartoon representation of the homology model of MOMP from *C. pneumoniae*, showing the location of the four variable domains (green). VS I, III and IV are in the barrel while the protease accessible VS II is in the extracellular space. *Right* - Cysteine residues are highlighted in blue. The N- and C-termini are coloured red and cyan, respectively.

**Figure 3:**
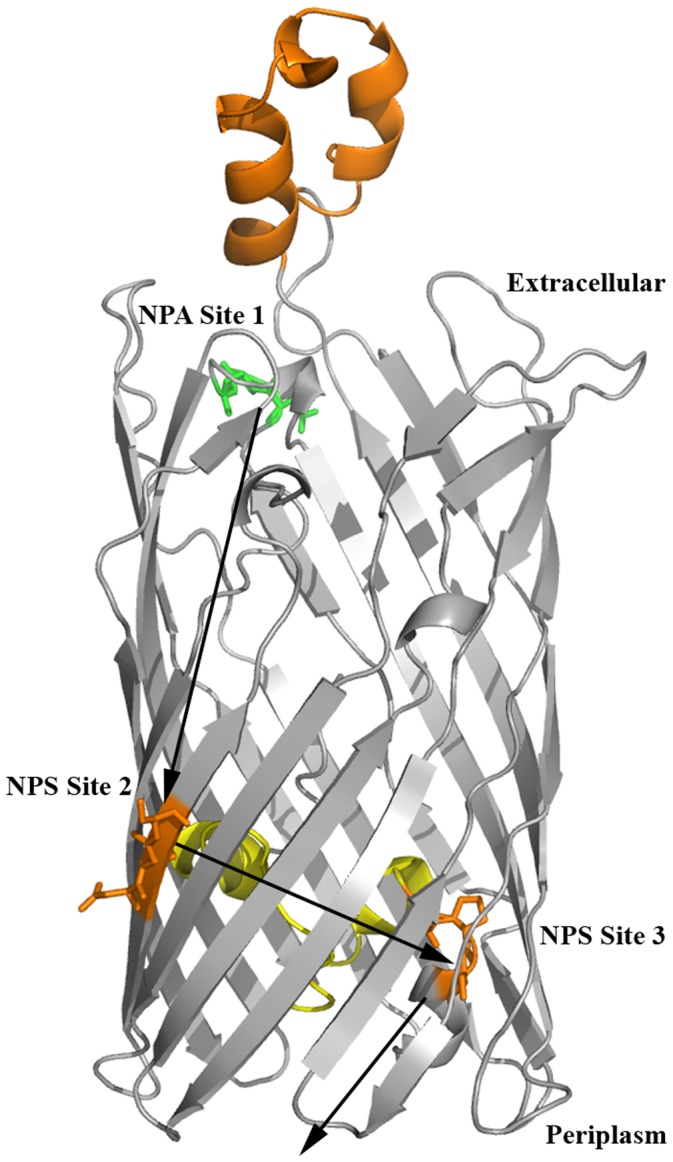
Hypothetical transport model of MOMP. The model shows the relative orientations of the NPA motif (green) and the two NPS motifs (orange) including the ‘hatch domain’ (yellow) and the proposed mechanism for solute recognition and transport (dashed lines). The NPA motif at site 1 is on the extracellular side and may serve to recognise and orient hydrophilic molecules for transport. The two NPS motifs are oriented at juxtaposition on the inside of the barrel wall (site 2 and site 3) to coordinate binding and release of ligands into the periplasm.

Both stereochemical and knowledgebase approaches were used to select and validate the template models, as revealed by Phyre and Intfold. First, each model was evaluated for stereochemical fitness by PROCHECK. Second, information regarding the availability of the variable segments of a MOMP homolog (from C. trachomatis) to proteolysis by trypsin was used as a guide to select an appropriate model [54]. And third, coordination in the location of the NPA motif and two NPS motifs of MOMP were utilised to reveal a plausible transport model. The final MOMP model (Figure 2), derived using MODELLER, reveals a 14 β-barrel protein with an external α-helix and a putative ‘hatch’ domain. The model indicates putative locations of the four variable domains and of the numerous cysteine residues. The resulting model provides a plausible transport model for *Chlamydophila* MOMP (Figure 3).

The final MOMP model yields favourable restraints in bond distances and dihedral angles, according to PROCHECK [31] with 87.2% of its residues favoured, 11.0% allowed, 1.3% generously allowed and 0.6% disallowed; G-factor of −0.5, and main chain bond length and angles of 96% and 86.4%, respectively. Assessment of the model for structural similarity, compared with the template, reveals an RMSD of 1.86 Å. The RMSD value corresponds to the geometric changes in the three dimensional structure between the model (MOMP) and the template (FadL).

### Comparison of Template MHC Alleles

**Figure 4:**
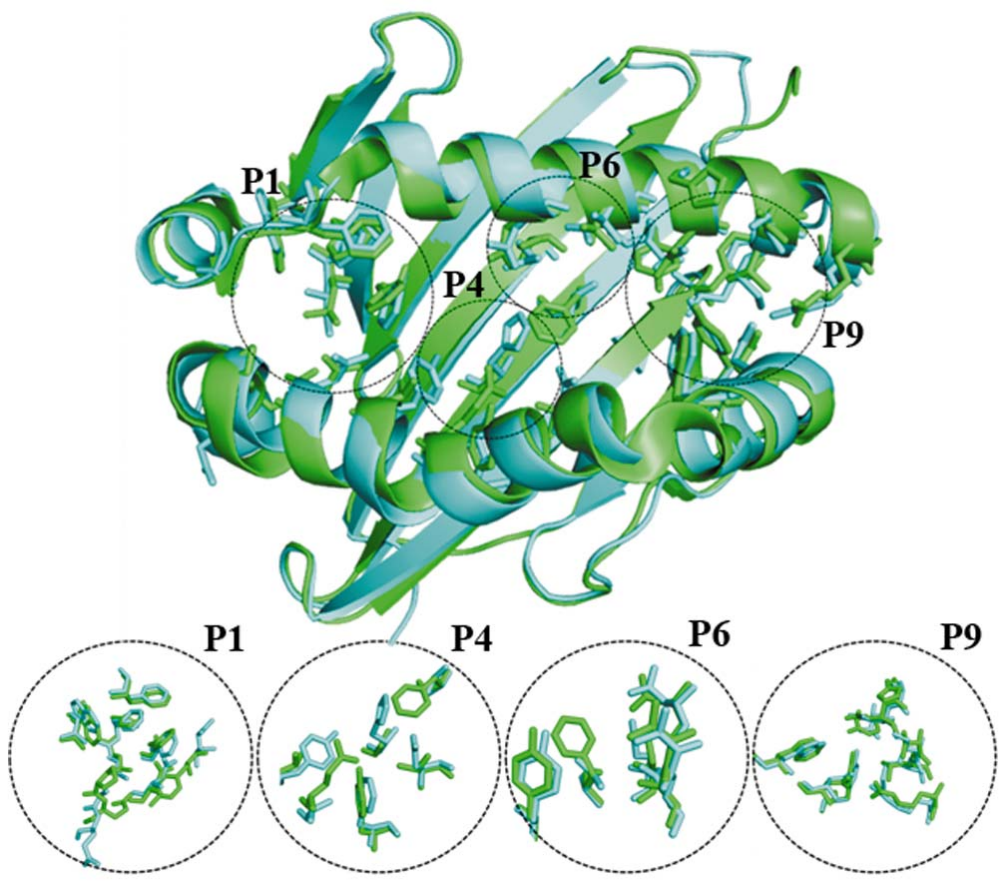
MHC II peptide binding pocket. Structure superposition of the binding sites of murine I-Ab (PBD code: 1MUJ) and human HLA-DR4 (PBD code: 2SEB). The binding pockets of the MHCs are designated P1, P4, P6 and P9. Pocket forming residues of I-Ab are coloured as green sticks and their corresponding HLA-DR4 residues, as identified by structural alignments, are coloured as cyan sticks.

Murine MHC I-Ab (PDB Code: 1MUJ) and its human ortholog (PDB Code: 2SEB) were used for the docking experiments. The MHC II proteins share a sequence identity of 56.4% and 78.8% similarity for the α-chain and, 64.6% identity and 84.1% similarity for the β-chain. Figure 4 shows the superposition of the two MHC II target proteins of interest sharing a structural identity of 1.24 Å. Similarly, the four most important hydrophobic pockets (labelled 1, 4, 6 and 9, Figure 4) reveal high sequence and structural similarity in amino acid composition and spatial orientation. Pocket 1 is the most highly conserved pocket between both alleles and largely dominated by aromatic amino acids of the α-chain. The αI31 in the human allele is substituted by leucine in the same position in the mouse allele. In contrast to pocket 1, pocket 4 is predominantly formed by the β-chain and has a very low amino acid identity between the two alleles. However, in each allele pocket 4 reveals a predominance of hydrophobic amino acids such as tyrosine, phenylalanine and valine. Negatively charged βD28, in the human allele, is substituted by a polar uncharged threonine residue in the mouse allele, whereas, human βA74 is occupied by a negatively charged glutamic acid residue in the mouse allele. More importantly, pocket 4 of the human allele possesses an autoimmune epitope ^67^LLEGKRAA^74^, occupied by ^67^ILERTRAE^74^ in the murine allele. The composition of pocket 6 is similar to that found in pocket 4; two negatively charged residues αE11 and αD66 are replaced by serine and valine, respectively. Pocket 9 appears to be the second most highly conserved pocket between the two alleles. There are substitutions of αA68 in the human allele for a positively charged histidine in the mouse allele and similarly, negatively charged βE9 in the human protein is substituted by tyrosine in the mouse protein. Taken together, there is higher hydrophobic character in pockets 1 and 9 than pockets 4 and 6, which appear to be mixed polar/non-polar in character.

### Molecular Docking MOMP-derived Peptides to MHC II

**Figure 5:**
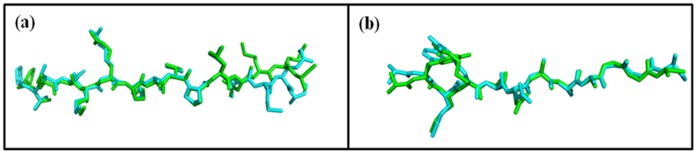
Docking validation. Results of poses derived from validation of the docking procedure showing (a) superposition of the docked pose from the extracted coordinates of human collagen II peptide and its native crystal state in I-Ab (PBD code: 1MUJ) and (b) superposition of the docked pose from the extracted coordinates of CLIP and its native crystal state in HLA DR4 (PBD code: 2SEB). The co-crystallised peptides are coloured green while the complementary docked peptides are shown in cyan.

Prior to the docking study of the novel MOMP-derived peptides, as validation of the docking procedure, coordinates of each of the known, bound peptides were extracted from the corresponding crystal structures (PDB Code: 1MUJ and 2SEB) and each were docked independently back into their respective MHC II target structures, without prior knowledge of the ligand binding site. In each case, the docked peptide was found bound to the target protein in a similar manner to the actual co-crystallised structure. For each peptide, the bound geometry was evaluated using calculated RMSD values between the docked ligand and the corresponding bound crystallographic structure. The resulting RMSDs were 0.97 Å and 0.51 Å for each of the two peptides extracted and docked into 1MUJ and 2SEB target structures, respectively (see Figure 5).

**Figure 6:**
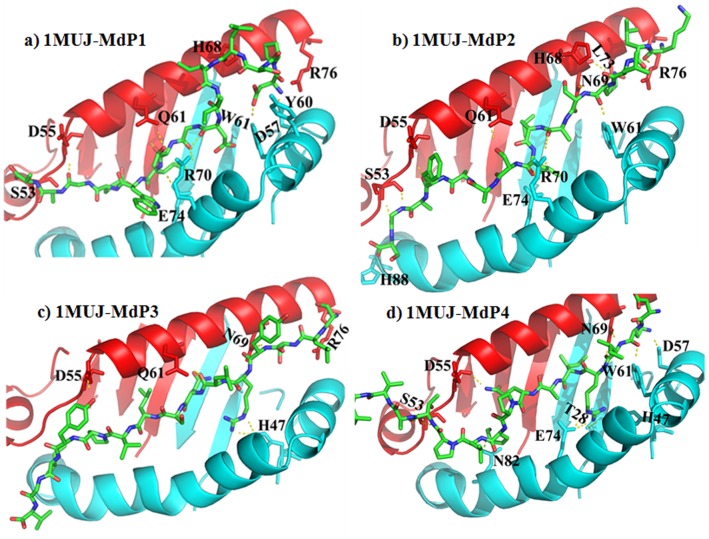
MOMP-derived peptide docking to I-Ab MCH II. Cartoon representations of the docked poses of four novel MOMP-derived peptides (MdP1-4, a–d) and the mouse I-Ab MCH II (PDB code 1MUJ). The peptides are coloured by element (green carbon; blue nitrogen; red oxygen). α-chains of the MHC II are coloured red, β-chains of the MHC II are coloured cyan. Yellow dashes represent potential hydrogen bonding.

**Figure 7:**
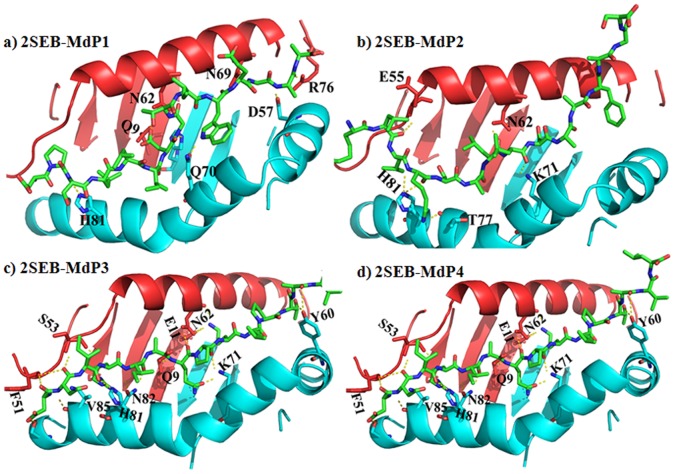
MOMP-derived peptide docking to HLA DR4 MCH II. Cartoon representations of the docked poses of four novel MOMP-derived peptides (MdP1-4, a–d) and the human HLA DR4 MHC II (PDB code 2SEB). The peptides are coloured by element (green carbon; blue nitrogen; red oxygen). α-chains of the MHC II are coloured red, β-chains of the MHC II are coloured cyan. Yellow dashes represent potential hydrogen bonding.

Molecular docking of four novel MOMP-derived peptides, postulated to exhibit anti-inflammatory activity against select MHC II targets, namely, mouse MHC class II I-Ab (PDB code: 1MUJ) and its human equivalent HLA-DR4 (PDB code: 2SEB), was performed in an effort to assess the specificity and mode of action of these peptides. The docked poses of each peptide are shown in Figures 6 and 7, for the mouse and human MHC II targets, respectively. The docking offers an explanation for participation of both electrostatic and hydrophobic forces driving the binding of these peptides to the MHC receptors. Consistent with the available literature, the electrostatic interaction is between the α-helices of the MHC and the backbone of the peptides. Among numerous interactions hydrogen bonds were identified to be consistent between residues of the peptides and αS53, αD55, αQ61, αR76, αN69 and βD57, βW61, βR70 and βE74 for 1MUJ. Residues αN62, αE55 and βK71, βH81 make hydrogen bond contacts with peptides docked onto HLA DR4. The side chains occupy solvent accessible pockets of the MHC with their aliphatic side chains. Aliphatic indices of the peptides shown in Table 1 shows close correlation with the number of van der Waal interactions in the hydrophobic pockets. These pockets are characterised by a clustering of hydrophobic residues which coordinate the orientation of peptide epitopes. Analysis of the molecular interactions between the peptides and the MHCs, as calculated using the programme CONTACTS, is provided in Tables S1 and S2. In general, there are more extensive interactions between these peptides and I-Ab than those observed in the HLA DR4 complexes. Analysis of the binding core of the peptides is complemented by online prediction using conventional immuno-informatic algorithms (Tables S3 and S4).

### Prediction of Peptide Binding Patterns

The binding pattern required to elicit an immune response via MHCs are well documented, therefore, a variety of prediction tools were used to determine the likely binding mode of each peptide sequence docked onto each of the two MHC receptors selected, using matrix or support vector based algorithms. In the first instance, the prediction accuracy of these tools was validated using the experimentally determined structures. These predictions are based on empirical results rendering a prediction of a 9-mer core binding peptide from the 15-mer peptide submitted. All except RANKPEP gives a single prediction of the most favoured binding core (refer to methods section for the list of other algorithms used). The binding pattern results, based on the predictions, were used to corroborate those adopted by the docked poses derived in the docking experiments. The resulting predictions are provided in Tables S3 and S4. Additional tools for the prediction of peptide binding to HLA DR4 were utilised, however, fewer methods were available suitably trained to render predictions for I-Ab.

## Discussion

### Homology Model for MOMP

The genus *Chlamydophila* is characterised by the presence of a cysteine rich outer membrane complex of proteins. More than 60% of the *Chlamydophila* outer membrane is comprised of a major outer membrane protein, MOMP, which over the years has been subject of intense research, largely targeted at vaccine development. These efforts appear to be driven by two major ideas. First, community acquired pneumonia is a serious public concern [55] and second, the consistency of association between *Chlamydophila pneumoniae* and cardiovascular events, such as atherosclerosis and stroke [55], suggest that vaccines against this pathogen may relieve atherosclerotic plaques [56]–[58]. However, engineered MOMP-derived oligopeptides capable of triggering the preferential elicitation of anti-atherogenic/inflammatory effects represents a new possibility for drug development.

Cysteine residues in mature MOMP constitute about 2% by residue. These cysteine residues have been proposed to maintain membrane integrity of the elementary bodies of the parasite by forming disulphide linkages with other outer membrane proteins [14], [59]. In addition, MOMP has been asserted to function as an energy nondependent β-barrel for the transport of solutes across the hydrophobic lipid cores of the outer membrane. Although previous work has provided extensive information on the immunological characteristics of *Chlamydophila* MOMP, in depth structural studies have been hampered due to complex protein aggregation and low level expression in bacteria [60]. Therefore, the use of computational methods presented herein has contributed to our current understanding of the complex structural and functional characteristics of MOMP.

In this study, a plausible homology model for the 40 kDa major outer membrane protein (MOMP) of *Chlamydophila pneumoniae* and molecular docking of MOMP-derived peptides to MHC class II molecules is presented. The three dimensional model of MOMP is based on the crystal structure of the fatty acid transporter of *E.coli*. The FadL of *E.coli* shares only 16.9% sequence identity but 39.55% similarity, the latter of which is sufficient for comparative modelling and hypothesis of protein function.

The MOMP model provides a plausible tertiary structure and putative functional mechanism for MOMP. The model suggests that several structurally important cysteine residues are located in regions predicted to be β-strands with their side chain residues facing outward, into the membrane bilayer. This lends support to the notion that MOMP exists in complex disulphide linkages with other *Chlamydophila* outer membrane proteins. Presumably, the location of these cysteines may not only enhance disulphide interaction but also provide attractive sites for fatty acid acylation. Furthermore, the structural rigidity of the membranes of *Chlamydophila,* attributed to MOMP, may involve favourable acylation by fatty acids within the interior of the lipid membrane. Interestingly, two inner membrane retention signals, namely ^49^CD and ^56^CD, are found within the first 57 residues of the immature MOMP protein. Although, current literature has supported sarkosyl insolubility and outer membrane localisation of MOMP in its native host *Chlamydophila*, there has been lack of evidence for the surface expression of MOMP in other expression hosts [61].

It has been suggested that differences in strain variability and antigenicity lie within four variable segments interspaced between five constant segments of MOMP. Some literature suggests that *Chlamydophila* variable segments are located on the outside of the outer membrane to enhance antigenicity, however, MOMP of *C. pneumoniae* is less immunologically dominant than that of *C. trachomatis* [62], which may mirror variation in the surface display of the antigenic variable segments. According to the homology model, three of the four variable segments are located in the β-strand region of the protein, which is presumably buried in the lipid membrane. Moreover, the second variable segment of MOMP is likely located in the hydrophobic alpha helix in the extracellular space (Figure 2). This location lends support to the experiments by Baehr et al., which demonstrated that variable segments II and IV are susceptible to protease treatment [63].

### A Transport Model for MOMP

The transport model hypothesised for MOMP is supported by the juxtaposition of two NPS motifs and a single NPA motif located on the extracellular surface of the protein (Figure3). The three motifs could represent independent substrate sites, which coordinate the transport of solutes across the membrane. A notable characteristic of the NPS motif in the barrel wall is the inward orientation of the hydrophobic proline ring anchor. The proposed model suggests that the NPA is positioned in the extracellular space to facilitate uptake of hydrophilic solutes whereas, the two NPS motifs may enhance the transport of a wide variety of hydrophobic solutes. Interestingly, members of the major intrinsic protein (MIP) family have similar characteristics. The aquaglyceroporin subgroup of MIPs exhibits the widest coverage of solute preference and may explain the substitution of alanine, in the canonical aquaporin NPA motif, by serine [64], [65]. The MOMP model presented herein supports the potential for MOMP to function as both a passive and substrate specific channel. The barrel orientation of the hydrophobic ring of proline could presumably anchor substrates at site 2, which could then be released into the periplasm by NPS residues at site 3. Alternatively, as in aquaporins, the energetically favoured extracellular NPA motif at site 1 may orient hydrophilic substrates directly into the barrel.

### MOMP-derived Peptides and Immune Response

It has been demonstrated, that MOMP from *C. pneumoniae* has anti-inflammatory, immunomodulatory and anti-atherosclerotic effects *in vivo* in C57Bl/6J apoE−/− mice [20]. This mice strain has a haplotype for the I-Ab MHC II. This work suggested that MOMP may play a role in *C. pneumoniae* modulation of macrophages and T cell responses, via the inhibition of antigen presentation and T cell co-stimulation. In an effort to understand such cell mediated immunomodulation by MOMP, four MOMP-derived peptides (formerly tested *in vivo*) were docked into the peptide binding pockets of mouse MHC II, I-Ab (PDB code 1MUJ) and its human ortholog HLA-DR4 (PDB code 2SEB). The molecular docking suggests a plausible mechanism through which MOMP-derived peptides may elicit an immune response via MHC binding.

This subtle immune evasiveness could be accomplished in at least one of five ways: i) short lived binding and rapid unloading of the MHC to avoid identifying bound peptides by TCR, ii) preferential orientation of short amino acid side chains to create an unfavourable contact with TCR. Such suboptimal binding topologies have been reported myelin basic protein/HLA-DR4 complex as a mechanism for escape from negative selection [66], iii) induction of an overwhelming protective signal by the resulting MHC-peptide complex, iv) stimulation of the generation of suppressive/regulatory T cell clones that dampens down inflammation through the synthesis and secretion of anti-inflammatory cytokines such as IL-10 and TGF beta [12] and, v) competitive binding of anti-inflammatory MOMP-derived peptides. Such a mechanism is supported by experiments, showing the effect of a single amino acid change in collagen II-derived peptide on loss of T cell activation, demonstrated by [67].

Murine and human MHC alleles were selected for computational analysis in this study, since I-Ab represents an already experimentally determined case study in the C57Bl/6J apoE−/− mice model [12]. Furthermore, I-Ab is bound to a CLIP peptide, which is a conserved step in the posttranslational processing of MHC proteins. The HLA-DR4 allele has an identified epitope for autoimmune disease susceptibility, which could be used to track the balance of the physiological effects of the docked peptides. Comparison of the residues, forming the binding pockets of the two MHCs (Figure 4), illustrate the presence of identical residues and residues sharing similar aliphatic side chains occupying strategic positions within the pockets. The highly homologous autoimmune susceptibility epitope lies between amino acids 67–74 of the β-chain of HLA-DR4. Interaction of bound peptides with Lysine 71 in the P4 pocket has been suggested to play a role in rheumatoid arthritis [68], [69]. Similarly, key residues within the epitope that indicate a strong susceptibility to diabetes and thyroiditis have been identified [70]. Structure based approaches have been used in the evaluation and design of potential vaccine candidates with success [71], [72]. Confidence in the docking procedure, as performed in this work on the MOMP-derived peptides, is supported by replication of the co-crystal structures of actual ligands in complex with the MHCs of interest (Figure 5). Three of the MOMP-derived peptides, demonstrated to have anti-inflammatory properties (peptides 2, 3 and 4), form potential hydrogen bond interactions with the notably βK71 residue of the human allele. Expectedly peptide 4, previously suggested to be the most likely candidate for vaccine development, is involved in two hydrogen bonds with βK71. However, this does not fully explain the anti-inflammatory properties of these peptides. It must be noted that the overall effect of binding and the complex molecular interaction between the peptides and the MHC must be consolidated in the variable antigenic display for TCR recognition. In the case of these docked peptides, extension of the aliphatic side chains were primarily in the clefts of the binding groove of the MHC. There appeared to be no significant bulges in the peptide 9-mer sequence, indicative of antigenic display, with the peptide docking in a buried fashion and each flanking residue slightly raised. This observation may be a function of the docking algorithm optimising MHC interactions, in the absence of a TCR partner, and therefore does not preclude the notion that these peptides may alter conformation upon subsequent TCR binding.

The docking experiments have indicated a preferred orientation of each peptide, when docked into the groove of the selected MHCs. Peptides 1, 2 and 3 were bound to murine MHC in an anti-parallel orientation compared with the binding pattern observed in the human allele; whereas peptide 4 bound the two alleles in a similar fashion. This in part could be due to the greater aliphatic indices of peptide 4 compared to peptides 1 and 2 (Table 1). Moreover, the formation of peptide-MHC complexes relies a great deal on the hydrophobic interactions between the specificity pockets of the MHC and aliphatic side chains of the peptide. Although this explanation may not account for the reverse orientations of bound peptide 3 seen in both alleles, flexibility of the MHC to bind peptides in multiple binding registers has been demonstrated (Tables S3 and S4), which leads to the possibility of more than one kind of immune response. This could be the origin of the mixed immunological response elicited by recombinant MOMP. Similarly, the same peptides can bind to MHC allotypes in different registers. The most important criterion for binding is conservation of the 9-mer binding core and establishment of a favourable MHC-TCR contact at the complementary determining regions. The docking results in this work are corroborated by the complementary immuno-informatic predictions and the evidence from immunisation studies using the selected MOMP peptides (Anne-Katrien Stark, PhD Thesis, University of Surrey, Guildford, United Kingdom, 2011). Immunisation of mice with peptides 3 and 4 induced CD4+ T cell regulation and a simultaneous decrease in the total CD4+ T cells compared to immunisation with peptides 1 and 2. Similarly, peptides 3 and 4 mouse immunization, monitored by elastin and collagen profiles of atheroma tissues, show that these peptides enhanced formation of more stable plaques than peptides 1 and 2. Taken together, these results provide support for the use of novel MOMP-derived peptides for further development as vaccine candidates for immune-therapy in the management of atherosclerosis and possibly other inflammatory diseases. Work is currently underway to assess the effect of these four peptides on T cell proliferation *in vitro*.

## Supporting Information

Figure S1 **Phylogenetic analyses of MOMP.** Phylogenetic maps of *Chlamydophila* and (a) aquaporins (b) aquaglyceroporins (c) long chain fatty acid transporters. Protein sequences were pooled from the UnitProt database based on the criteria to possess NPA and NPA motifs. The phylogenetic maps were rendered using TreeView.
(TIF)

Table S1 **Hydrogen bond and van der Waals contacts for the four novel MOMP-derived peptides (MdPs) docked into the peptide binding pocket of the α and β chains of the murine I-Ab MHC II protein (PBD code 1MUJ).**
(DOCX)

Table S2 **Hydrogen bond and van der Waals contacts for the four novel MOMP-derived peptides (MdPs) docked into the peptide binding pocket of the α and β chains of the human HLA DR4 MHC II protein (PBD code 2SEB).**
(DOCX)

Table S3 **Prediction of the binding pattern of four novel MOMP derived peptide to murine MHC class II, I-Ab (PBD code 1MUJ), as determined using online servers.**
(DOCX)

Table S4 **Prediction of the binding pattern of four novel MOMP-derived peptides to human HLA DR4 MHC II protein (PBD code 2SEB), as determined using online servers.**
(DOCX)
