# Supplementary figures and images for: A Novel Transport Mechanism for MOMP in Chlamydophila pneumoniae and Its Putative Role in Immune-Therapy
Source: PLoS One. 2013 Apr 24;8(4):e61139. doi: 10.1371/journal.pone.0061139 (PMC3634821; doi:10.1371/journal.pone.0061139)

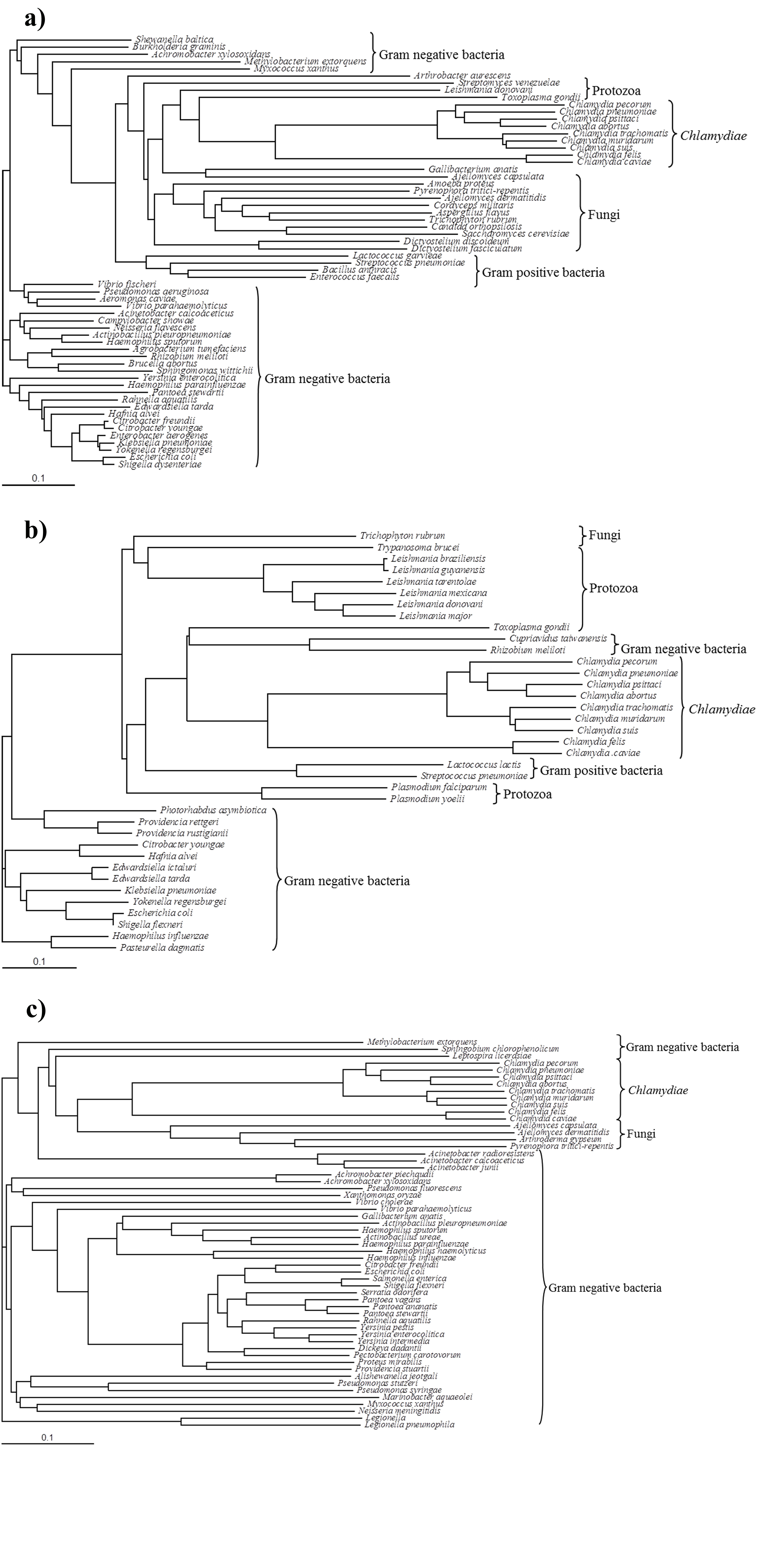

Supplement: Figure S1 — Phylogenetic analyses of MOMP. Phylogenetic maps of Chlamydophila and (a) aquaporins (b) aquaglyceroporins (c) long chain fatty acid transporters. Protein sequences were pooled from the UnitProt database based on the criteria to possess NPA and NPA motifs. The phylogenetic maps were rendered using TreeView. (TIF) [file pone.0061139.s001.tif]
