# Supplementary material for: A Novel Transport Mechanism for MOMP in Chlamydophila pneumoniae and Its Putative Role in Immune-Therapy
Source: PLoS One. 2013 Apr 24;8(4):e61139. doi: 10.1371/journal.pone.0061139 (PMC3634821; doi:10.1371/journal.pone.0061139)
Supplement: Table S1 — Hydrogen bond and van der Waals contacts for the four novel MOMP-derived peptides (MdPs) docked into the peptide binding pocket of the α and β chains of the murine I-Ab MHC II protein (PBD code 1MUJ). (DOCX) [file pone.0061139.s002.docx]

**Table S1.** Hydrogen bond and van der Waals contacts for the four novel MOMP-derived peptides (MdPs) docked into the peptide binding pocket of the α and β chains of the murine I-Ab MHC II protein (PBD code 1MUJ).

|  | **H-bond interactions with distances*** | | | |  | |  |  |
| --- | --- | --- | --- | --- | --- | --- | --- | --- |
| **Peptide** | **α chain** | **Distance Å** | **β chain** | **Distance Å** | | **Number of favourable vdW interactions** | **Number of unfavourable vdW interactions** | **Total number of vdW interactions** |
| **MdP1** | Asp 1C N ... Arg 76A NH2 | 3.23 | Asp 1C N ... Asp 57B OD1 | 2.93 | | 43 | 9 | 52 |
|  | Leu 4C O ... His 68A NE2 | 2.70 | Asp 1C OD2 ... Trp 61B NE1 | 2.93 | |  |  |  |
|  | Thr 9C N ... Gln 61A NE2 | 3.26 | Ser 3C OG ... Tyr 60B OH | 2.85 | |  |  |  |
|  | Thr 9C O ... Asn 62A ND2 | 3.15 | Asp 7C O ... Arg 70B NH2 | 3.02 | |  |  |  |
|  | Thr 9C OG1 ... Gln 61A NE2 | 3.08 | Asp 7C OD1 ... Trp 61B NE1 | 3.17 | |  |  |  |
|  | Gly 13C O ... Asp 55A N | 2.91 | Gly 8C O ... Arg 70B NH2 | 2.69 | |  |  |  |
|  | Ala 15C N ... Ser 53A OG | 3.23 | Thr 9C OG1 ... Arg 70B NH2 | 3.09 | |  |  |  |
|  | Ala 15C OXT ... Ser 53A OG | 2.89 | Thr 9C OG1 ... Arg 70B NH1 | 2.57 | |  |  |  |
|  |  |  | Ile 10C N ... Glu 74B OE2 | 2.85 | |  |  |  |
|  |  |  |  |  | |  |  |  |
| **MdP2** | Lys 1C O ... Arg 76A NE | 3.14 | Lys 4C O ... Trp 61B NE1 | 2.99 | | 69 | 12 | 81 |
|  | Lys 1C O ... Arg 76A NH2 | 2.90 | Ala 6C O ... Arg 70B NH2 | 3.11 | |  |  |  |
|  | Leu 3C O ... His 68A NE2 | 3.03 | Leu 8C O ... Arg 70B NH2 | 2.71 | |  |  |  |
|  | Lys 4C NZ ... Asn 69A O | 3.20 | Leu 8C O ... Arg 70B NE | 2.24 | |  |  |  |
|  | Lys 4C NZ ... Leu 73A N | 3.08 | Leu 8C O ... Glu 74B OE2 | 2.67 | |  |  |  |
|  | Ser 5C N ... Asn 69A OD1 | 2.95 | Ser 15C OXT... His 88B ND1 | 3.27 | |  |  |  |
|  | Ser 5C O ... Asn 69A ND2 | 3.07 |  |  | |  |  |  |
|  | Ser 5C O ... Asn 69A OD1 | 3.21 |  |  | |  |  |  |
|  | Ser 9C OG ... Gln 61A OE1 | 2.95 |  |  | |  |  |  |
|  | Phe 12C O ... Asp 55A N | 3.00 |  |  | |  |  |  |
|  | Gly 14C N ... Ser 53A O | 2.79 |  |  | |  |  |  |
|  | Gly 14C O ... Ser 53A N | 3.00 |  |  | |  |  |  |
|  |  |  |  |  | |  |  |  |
| **MdP3** | Ser 1C O ... Arg 76A NE | 3.09 | Ser 3C OG ... Asp 57B OD1 | 3.17 | | 76 | 4 | 80 |
|  | Ser 1C O... Arg 76A NH2 | 2.88 | Arg 5C NE ... Tyr 30B OH | 2.63 | |  |  |  |
|  | Tyr 4C N ... His 68A NE2 | 3.14 | Arg 5C NE ... His 47B NE2 | 3.18 | |  |  |  |
|  | Tyr 4C N ... Asn 69A OD1 | 3.08 | Arg 5C NH1 ...Tyr 30B OH | 3.16 | |  |  |  |
|  | Tyr 4C O ... Asn 69A OD1 | 2.61 | Arg 5C NH1 ... His 47B NE2 | 3.12 | |  |  |  |
|  | Leu 6C O ... Gln 61A NE2 | 3.08 | Arg 5C NH1... Thr 71B OG1 | 2.89 | |  |  |  |
|  | Asn 7C OD1... Asn 62A OD1 | 2.63 | Ser 8C OG ... Arg 70B NE | 3.14 | |  |  |  |
|  | Asn 7C OD1... Asn 62A O | 3.27 | Ser 8C OG ... Arg 70B NH1 | 3.20 | |  |  |  |
|  | Tyr 12C OH ... Asp 55A OD2 | 2.99 |  |  | |  |  |  |
|  |  |  |  |  | |  |  |  |
| **MdP4** | Asp 1C O ... His 68A NE2 | 2.77 | Asn 2C O ... Trp 61B NE1 | 2.86 | | 60 | 12 | 72 |
|  | Asp 1C OD1 ... His 68A NE2 | 3.14 | Asn 2C ND2... Asp 57B OD2 | 3.22 | |  |  |  |
|  | Asn 2C ND2... Arg 76A NH2 | 2.85 | Asn 2C ND2... Asp 57B OD1 | 2.87 | |  |  |  |
|  | Asn 2C OD1... Asn 69A O | 3.29 | Arg 4C NE ...Thr 71B OG1 | 2.82 | |  |  |  |
|  | Ile 3C N ... Asn 69A OD1 | 2.97 | Arg 4C NE ... Glu 74B OE1 | 2.82 | |  |  |  |
|  | Ile 3C O ... Asn 69A ND2 | 2.96 | Arg 4C NH1... Thr 28B OG1 | 3.18 | |  |  |  |
|  | Ala 6C O ... Gln 61A NE2 | 3.03 | Arg 4C NH1...Glu 74B OE1 | 3.04 | |  |  |  |
|  | Ala 6C O ... Gln 61A OE1 | 3.08 | Arg 4C NH2...Tyr 30B OH | 2.99 | |  |  |  |
|  | Gln 7C NE2 ... Asp 55A O | 3.00 | Arg 4C NH2 ...His 47B NE2 | 2.91 | |  |  |  |
|  | Gln 7C NE2 ... Gly 58A N | 3.22 | Arg 4C NH2... Thr 71B OG1 | 2.91 | |  |  |  |
|  | Thr 12C N ... Ser 53A OG | 3.05 | Lys 9C NZ... Asn 82B ND2 | 3.17 | |  |  |  |
|  | Thr 12C O ... Ser 53A OG | 2.90 | Lys 9C NZ... Asn 82B OD1 | 3.02 | |  |  |  |
|  | Thr 12C OG1 ... Ser 53A OG | 2.55 |  |  | |  |  |  |
|  | Thr 12C OG1 ... Phe 54A N | 3.00 |  |  | |  |  |  |

*Hydrogen bonds were assigned if the distance between two electronegative atoms was < 3.3 Å and van der Waals interactions if the separation between non-bonded atoms was < 4.0 Å.
