# Supplementary material for: A Novel Transport Mechanism for MOMP in Chlamydophila pneumoniae and Its Putative Role in Immune-Therapy
Source: PLoS One. 2013 Apr 24;8(4):e61139. doi: 10.1371/journal.pone.0061139 (PMC3634821; doi:10.1371/journal.pone.0061139)
Supplement: Table S2 — Hydrogen bond and van der Waals contacts for the four novel MOMP-derived peptides (MdPs) docked into the peptide binding pocket of the α and β chains of the human HLA DR4 MHC II protein (PBD code 2SEB). (DOCX) [file pone.0061139.s003.docx]

**Table S2.** Hydrogen bond and van der Waals contacts for the four novel MOMP-derived peptides (MdPs) docked into the peptide binding pocket of the α and β chains of the human HLA DR4 MHC II protein (PBD code 2SEB).

|  | **H-bond interactions with distances*** | | | | | |  | |  |  |
| --- | --- | --- | --- | --- | --- | --- | --- | --- | --- | --- |
| **Peptide** | **α chain** | | **Distance Å** | | **β chain** | **Distance Å** | | **Number of favourable vdW interactions** | **Number of unfavourable vdW interactions** | **Total number of vdW interactions** |
| **MdP1** | Asp 7C OD2 ... Gln 9A NE2 | 3.14 | | Ser 3C N ... His 81B NE2 | | 3.07 | | 51 | 8 | 59 |
|  | Asp 7C OD2... Asn 62A ND2 | 2.87 | | Trp 11C NE1...Gln 70B OE1 | | 2.75 | |  |  |  |
|  | Gly 8C O ... Asn 62A ND2 | 3.27 | | Ala 14C N ... Asp 57B OD1 | | 2.67 | |  |  |  |
|  | Gly 8C O ... Asn 62A OD1 | 2.61 | |  | |  | |  |  |  |
|  | Gly 13C ... Asn 69A OD1 | 3.26 | |  | |  | |  |  |  |
|  | Ala 14C O ... Arg 76A NH1 | 2.92 | |  | |  | |  |  |  |
|  |  |  | |  | |  | |  |  |  |
| **MdP2** | Leu 2C O ... Glu 55A N | 3.23 | | Lys 4C N ... His 81B NE2 | | 3.28 | | 34 | 5 | 39 |
|  | Leu 7C O ... Asn 62A ND2 | 3.09 | | Lys 4C O ... His 81B NE2 | | 2.79 | |  |  |  |
|  |  |  | | Lys 4C NZ ... Thr 77B O | | 2.94 | |  |  |  |
|  |  |  | | Leu 8C O ... Lys 71B NZ | | 2.76 | |  |  |  |
|  |  |  | |  | |  | |  |  |  |
| **MdP3** | Ser 1C O ... Glu 55A N | 2.88 | | Arg 5C NE ... His 81B NE2 | | 3.27 | | 41 | 7 | 48 |
|  | Tyr 4C OH ... Gln 57A NE2 | 2.78 | | Arg 5C NH1... Thr 77B O | | 2.89 | |  |  |  |
|  | Asn 7C ND2... Gln 9A NE2 | 3.25 | | Arg 5C NH1... His 81B ND1 | | 3.22 | |  |  |  |
|  | Asn 7C ND2 ... Gln 9A OE1 | 2.96 | | Arg 5C NH2 ... His 81B NE2 | | 3.27 | |  |  |  |
|  |  |  | | Arg 5C NH2 ... Thr 77B O | | 2.84 | |  |  |  |
|  |  |  | | Asn 7C O ... Lys 71B NZ | | 2.84 | |  |  |  |
|  |  |  | | Leu 9C O ... Gln 70B NE2 | | 2.75 | |  |  |  |
|  |  |  | | Leu 9C O ... Gln 70B OE1 | | 2.94 | |  |  |  |
|  |  |  | | Ile 13C O ... Tyr 60B OH | | 2.90 | |  |  |  |
|  |  |  | |  | |  | |  |  |  |
| **MdP4** | Asp 1C N ... Phe 51A O | 2.87 | | Asp 1C N ... Val 85B O | | 2.77 | | 77 | 8 | 85 |
|  | Asp 1C O ... Phe 51A O | 2.61 | | Ile 3C O ... His 81B NE2 | | 3.23 | |  |  |  |
|  | Asn 2C O ... Ser 53A N | 2.99 | | Ile 5C N ... Asn 82B OD1 | | 2.95 | |  |  |  |
|  | Arg 4C N ... Ser 53A O | 2.93 | | Ile 5C O ... Asn 82B ND2 | | 3.03 | |  |  |  |
|  | Gln 7C N ... Gln 9A OE1 | 2.97 | | Gln 7C OE1 ... Lys 71B NZ | | 2.84 | |  |  |  |
|  | Gln 7C O ... Asn 62A ND2 | 3.11 | | Pro 8C O ... Lys 71B NZ | | 2.56 | |  |  |  |
|  | Lys 9C NZ ... Asn 62A OD1 | 2.74 | | Ala 13C N ... Tyr 60B OH | | 3.02 | |  |  |  |
|  | Lys 9C NZ ... Glu 11A OE2 | 2.98 | |  | |  | |  |  |  |
|  | Lys 9C NZ ... Asn 62A O | 2.85 | |  | |  | |  |  |  |
|  | Lys 9C NZ ... Asp 66A OD1 | 3.24 | |  | |  | |  |  |  |

*Hydrogen bonds were assigned if the distance between two electronegative atoms was < 3.3 Å and van der Waals interactions if the separation between non-bonded atoms was < 4.0 Å
