# Supplementary material for: A Novel Transport Mechanism for MOMP in Chlamydophila pneumoniae and Its Putative Role in Immune-Therapy
Source: PLoS One. 2013 Apr 24;8(4):e61139. doi: 10.1371/journal.pone.0061139 (PMC3634821; doi:10.1371/journal.pone.0061139)
Supplement: Table S3 — Prediction of the binding pattern of four novel MOMP derived peptide to murine MHC class II, I-Ab (PBD code 1MUJ), as determined using online servers. (DOCX) [file pone.0061139.s004.docx]

**Table S3.** Prediction of the binding pattern of four novel MOMP derived peptide to murine MHC class II, I-Ab (PBD code 1MUJ), as determined using online servers.

| **MdP1-1MUJ**  DPSLLIDGTIWEGAA | | **Peptide register** | | | | | | | | | | | | | | | | | | | | |
| --- | --- | --- | --- | --- | --- | --- | --- | --- | --- | --- | --- | --- | --- | --- | --- | --- | --- | --- | --- | --- | --- | --- |
| **Prediction method** | **Binding core** | **-6** | **-5** | **-4** | **-3** | **-2** | **-1** | **1** | **2** | **3** | **4** | **5** | **6** | **7** | **8** | **9** | **+1** | **+2** | **+3** | **+4** | **+5** | **+6** |
| NetMHCII server | LIDGTIWEG |  |  | D | P | S | L | L | I | D | G | T | I | W | E | G | A | A |  |  |  |  |
| SMM_align | SLLIDGTIW |  |  |  |  | D | P | S | L | L | I | D | G | T | I | W | E | G | A | A |  |  |
| NN_align | LIDGTIWEG |  |  | D | P | S | L | L | I | D | G | T | I | W | E | G | A | A |  |  |  |  |
| RANKPEP server | DGTIWEGAA | D | P | S | L | L | I | D | G | T | I | W | E | G | A | A |  |  |  |  |  |  |
| MHC2PRED server | PSLLIDGTI |  |  |  |  |  | D | P | S | L | L | I | D | G | T | I | W | E | G | A | A |  |
| **MdP2-1MUJ**  KLLKSALLSAAFAGS | | | | | | | | | | | | | | | | | | | | | |  |
| **Prediction method** | **Binding core** | **-6** | **-5** | **-4** | **-3** | **-2** | **-1** | **1** | **2** | **3** | **4** | **5** | **6** | **7** | **8** | **9** | **+1** | **+2** | **+3** | **+4** | **+5** | **+6** |
| NetMHCII server | LKSALLSAA |  |  |  |  | K | L | L | K | S | A | L | L | S | A | A | F | A | G | S |  |  |
| SMM_align | LKSALLSAA |  |  |  |  | K | L | L | K | S | A | L | L | S | A | A | F | A | G | S |  |  |
| NN_align | LKSALLSAA |  |  |  |  | K | L | L | K | S | A | L | L | S | A | A | F | A | G | S |  |  |
| RANKPEP server | LKSALLSAA |  |  |  |  | K | L | L | K | S | A | L | L | S | A | A | F | A | G | S |  |  |
| MHC2PRED server | KLLKSALLS |  |  |  |  |  |  | K | L | L | K | S | A | L | L | S | A | A | F | A | G | S |
|  |  |  |  |  |  |  |  |  |  |  |  |  |  |  |  |  |  |  |  |  |  |  |
| **MdP3-1MUJ**  SLSYRLNSLVPYIGV | | | | | | | | | | | | | | | | | | | | | | |
| **Prediction method** | **Binding core** | **-6** | **-5** | **-4** | **-3** | **-2** | **-1** | **1** | **2** | **3** | **4** | **5** | **6** | **7** | **8** | **9** | **+1** | **+2** | **+3** | **+4** | **+5** | **+6** |
| NetMHCII server | YRLNSLVPY |  |  |  | S | L | S | Y | R | L | N | S | L | V | P | Y | I | G | V |  |  |  |
| SMM_align | YRLNSLVPY |  |  |  | S | L | S | Y | R | L | N | S | L | V | P | Y | I | G | V |  |  |  |
| NN_align | YRLNSLVPY |  |  |  | S | L | S | Y | R | L | N | S | L | V | P | Y | I | G | V |  |  |  |
| RANKPEP server | SYRLNSLVP |  |  |  |  | S | L | S | Y | R | L | N | S | L | V | P | Y | I | G | V |  |  |
| MHC2PRED server | YRLNSLVPY |  |  |  | S | L | S | Y | R | L | N | S | L | V | P | Y | I | G | V |  |  |  |
| **MdP4-1MUJ**  DNIRIAQPKLPTAVL | | | | | | | | | | | | | | | | | | | | | | |
| **Prediction method** | **Binding core** | **-6** | **-5** | **-4** | **-3** | **-2** | **-1** | **1** | **2** | **3** | **4** | **5** | **6** | **7** | **8** | **9** | **+1** | **+2** | **+3** | **+4** | **+5** | **+6** |
| NetMHCII server | AQPKLPTAV |  | D | N | I | R | I | A | Q | P | K | L | P | T | A | V | L |  |  |  |  |  |
| SMM_align | AQPKLPTAV |  | D | N | I | R | I | A | Q | P | K | L | P | T | A | V | L |  |  |  |  |  |
| NN_align | AQPKLPTAV |  | D | N | I | R | I | A | Q | P | K | L | P | T | A | V | L |  |  |  |  |  |
| RANKPEP server | IAQPKLPTA |  |  | D | N | I | R | I | A | Q | P | K | L | P | T | A | V | L |  |  |  |  |
| MHC2PRED server | RIAQPKLPT |  |  |  | D | N | I | R | I | A | Q | P | K | L | P | T | A | V | L |  |  |  |
