# Supplementary material for: A Novel Transport Mechanism for MOMP in Chlamydophila pneumoniae and Its Putative Role in Immune-Therapy
Source: PLoS One. 2013 Apr 24;8(4):e61139. doi: 10.1371/journal.pone.0061139 (PMC3634821; doi:10.1371/journal.pone.0061139)
Supplement: Table S4 — Prediction of the binding pattern of four novel MOMP-derived peptides to human HLA DR4 MHC II protein (PBD code 2SEB), as determined using online servers. (DOCX) [file pone.0061139.s005.docx]

**Table S4.** Prediction of the binding pattern of four novel MOMP-derived peptides to human HLA DR4 MHC II protein (PBD code 2SEB), as determined using online servers.

| **MdP1-2SEB**  DPSLLIDGTIWEGAA | | **Peptide register** | | | | | | | | | | | | | | | | | | | |
| --- | --- | --- | --- | --- | --- | --- | --- | --- | --- | --- | --- | --- | --- | --- | --- | --- | --- | --- | --- | --- | --- |
| **Prediction method** | **Binding core** | **-5** | **-4** | **-3** | **-2** | **-1** | **1** | **2** | **3** | **4** | **5** | **6** | **7** | **8** | **9** | **+1** | **+2** | **+3** | **+4** | **+5** | **+6** |
| NetMHCII server | LLIDGTIWE |  |  | D | P | S | L | L | I | D | G | T | I | W | E | G | A | A |  |  |  |
| SMM_align | LLIDGTIWE |  |  | D | P | S | L | L | I | D | G | T | I | W | E | G | A | A |  |  |  |
| NN_align | LLIDGTIWE |  |  | D | P | S | L | L | I | D | G | T | I | W | E | G | A | A |  |  |  |
| STURNIOLO | LLIDGTIWE |  |  | D | P | S | L | L | I | D | G | T | I | W | E | G | A | A |  |  |  |
| RANKPEP server | LLIDGTIWE |  |  | D | P | S | L | L | I | D | G | T | I | W | E | G | A | A |  |  |  |
| MHC2PRED server | DPSLLIDGT |  |  |  |  |  | D | P | S | L | L | I | D | G | T | I | W | E | G | A | A |
| PROPRED server | LLIDGTIWE |  |  | D | P | S | L | L | I | D | G | T | I | W | E | G | A | A |  |  |  |
| SVMHC server | LLIDGTIWE |  |  | D | P | S | L | L | I | D | G | T | I | W | E | G | A | A |  |  |  |
| **MdP2-2SEB**  KLLKSALLSAAFAGS | | | | | | | | | | | | | | | | | | | | | |
| **Prediction method** | **Binding core** | **-5** | **-4** | **-3** | **-2** | **-1** | **1** | **2** | **3** | **4** | **5** | **6** | **7** | **8** | **9** | **+1** | **+2** | **+3** | **+4** | **+5** | **+6** |
| NetMHCII server | LKSALLSAA |  |  |  | K | L | L | K | S | A | L | L | S | A | A | F | A | G | S |  |  |
| SMM_align | LKSALLSAA |  |  |  | K | L | L | K | S | A | L | L | S | A | A | F | A | G | S |  |  |
| NN_align | LKSALLSAA |  |  |  | K | L | L | K | S | A | L | L | S | A | A | F | A | G | S |  |  |
| STURNIOLO | LLKSALLSA |  |  |  |  | K | L | L | K | S | A | L | L | S | A | A | F | A | G | S |  |
| RANKPEP server | LLKSALLSA |  |  |  |  | K | L | L | K | S | A | L | L | S | A | A | F | A | G | S |  |
| MHC2PRED server | SALLSAAFA |  | K | L | L | K | S | A | L | L | S | A | A | F | A | G | S |  |  |  |  |
| PROPRED server | LLKSALLSA |  |  |  |  | K | L | L | K | S | A | L | L | S | A | A | F | A | G | S |  |
| SVMHC server | LLKSALLSA |  |  |  |  | K | L | L | K | S | A | L | L | S | A | A | F | A | G | S |  |
| **MdP3-2SEB**  SLSYRLNSLVPYIGV | | | | | | | | | | | | | | | | | | | | | |
| **Prediction method** | **Binding core** | **-5** | **-4** | **-3** | **-2** | **-1** | **1** | **2** | **3** | **4** | **5** | **6** | **7** | **8** | **9** | **+1** | **+2** | **+3** | **+4** | **+5** | **+6** |
| NetMHCII server | YRLNSLVPY |  |  | S | L | S | Y | R | L | N | S | L | V | P | Y | I | G | V |  |  |  |
| SMM_align | LNSLVPYIG | S | L | S | Y | R | L | N | S | L | V | P | Y | I | G | V |  |  |  |  |  |
| NN_align | YRLNSLVPY |  |  | S | L | S | Y | R | L | N | S | L | V | P | Y | I | G | V |  |  |  |
| STURNIOLO | YRLNSLVPY |  |  | S | L | S | Y | R | L | N | S | L | V | P | Y | I | G | V |  |  |  |
| RANKPEP server | SYRLNSLVP |  |  |  | S | L | S | Y | R | L | N | S | L | V | P | Y | I | G | V |  |  |
| MHC2PRED server | LSYRLNSLV |  |  |  |  | S | L | S | Y | R | L | N | S | L | V | P | Y | I | G | V |  |
| PROPRED server | YRLNSLVPY |  |  | S | L | S | Y | R | L | N | S | L | V | P | Y | I | G | V |  |  |  |
| SVMHC server | YRLNSLVPY |  |  | S | L | S | Y | R | L | N | S | L | V | P | Y | I | G | V |  |  |  |
| **MdP4-2SEB**  DNIRIAQPKLPTAVL | | | | | | | | | | | | | | | | | | | | | |
| **Prediction method** | **Binding core** | **-5** | **-4** | **-3** | **-2** | **-1** | **1** | **2** | **3** | **4** | **5** | **6** | **7** | **8** | **9** | **+1** | **+2** | **+3** | **+4** | **+5** | **+6** |
| NetMHCII server | IRIAQPKLP |  |  |  | D | N | I | R | I | A | Q | P | K | L | P | T | A | V | L |  |  |
| SMM_align | IAQPKLPTA |  | D | N | I | R | I | A | Q | P | K | L | P | T | A | V | L |  |  |  |  |
| NN_align | IRIAQPKLP |  |  |  | D | N | I | R | I | A | Q | P | K | L | P | T | A | V | L |  |  |
| STURNIOLO | IRIAQPKLP |  |  |  | D | N | I | R | I | A | Q | P | K | L | P | T | A | V | L |  |  |
| RANKPEP server | RIAQPKLPT |  |  | D | N | I | R | I | A | Q | P | K | L | P | T | A | V | L |  |  |  |
| MHC2PRED server | IRIAQPKLP |  |  |  | D | N | I | R | I | A | Q | P | K | L | P | T | A | V | L |  |  |
| PROPRED server | IRIAQPKLP |  |  |  | D | N | I | R | I | A | Q | P | K | L | P | T | A | V | L |  |  |
| SVMHC server | IRIAQPKLP |  |  |  | D | N | I | R | I | A | Q | P | K | L | P | T | A | V | L |  |  |
